# The BELANA questionnaire measures the health economic burdens of food allergy and intolerance in multinational settings

**DOI:** 10.1186/2045-7022-1-S1-P60

**Published:** 2011-08-12

**Authors:** Norbert Rösch, Sabine Schnadt, Andreas Arens-Volland, Frank Feidert, Xavier Miller, Sarah Kohler, Volker König, Petra Schmalz, Ralhp Mösges

**Affiliations:** 1 Centre de Recherche Public Henri Tudor, SANTEC (GA2LEN Collaborating Centre), Luxembourg, Luxembourg; 2 German Allergy and Asthma Association, Moenchengladbach, Germany; 3 Centre Hospitalier de Luxembourg, ORL-Eich, Luxembourg, Luxembourg; 4 Luxembourg Society of Dermato-Venereology, Luxembourg, Luxembourg; 5 University Hospital of Cologne, IMSIE, Cologne, Germany

## Background

The Health economic burdens of patients with food allergies or intolerance and their Health Related Quality of Life (HR-QoL) are unknown in Luxembourg and most other countries. Within the Luxembourg MENSSANA project, a new approach has been defined to evaluate health economic effects in a multinational setting.

## Methods

The BELANA questionnaire (Burdens and Expenses of Living as Adult with Nutrition based Allergy or Intolerance) has been developed to measure the temporal and financial burdens from the patient’s perspective. BELANA includes possible temporal losses (disability days, reduction in productivity, hospitalizations) as well as direct costs (additional costs for food purchases). For the complementary measurement of Health Related Quality of Life (HR-QoL), a combined appliance of the disease-specific FAQLQ-AF (Food Allergy Quality of Life Questionnaire - Adult Form) and the generic SF-12v1 has been chosen. BELANA avoids questions, already included in the standard HR-QoL questionnaires.

## Results

BELANA has been tested in a web-based pilot survey with 51 adults (age >18). The patient's acceptance has been confirmed by the number of completed questionnaires (50 of 51) and the high response rates for health-economic items (76% to 100%). The combination of BELANA, FAQLQ-AF and SF-12v1 has been completed in an average of 22 minutes. An age-related selection bias has not been confirmed (median age: 37.9 years, range from 19 to 72 years, SD = 12.4 years). To minimise possible seasonal effects BELANA is further used in a one-year longitudinal study with 314 patients. According to an interim evaluation, most patient's expenses are linked to the purchase of dietary foods.

## Conclusions

We continue to utilize BELANA as a useful tool for the evaluation of health economic burdens of food allergy and intolerance. BELANA, coupled with the benefits of electronic data collection, offers real time plausibility checks by limiting the workload for survey completion and data evaluation.

